# Lack of knowledge and misperceptions about thalassaemia among college students in Bangladesh: a cross-sectional baseline study

**DOI:** 10.1186/s13023-020-1323-y

**Published:** 2020-02-21

**Authors:** Mohammad Sorowar Hossain, Md. Mahbub Hasan, Enayetur Raheem, Muhammad Sougatul Islam, Abdullah Al Mosabbir, Mary Petrou, Paul Telfer, Mahbubul H. Siddiqee

**Affiliations:** 1 Biomedical Research Foundation, Dhaka, Bangladesh; 2 Independent University, Bangladesh (IUB), Dhaka, Bangladesh; 3 Bangladesh University of Health Sciences, Dhaka, Bangladesh; 4 Department of Genetic Engineering and Biotechnology, University of Chittagong, Chattogram 4331, Bangladesh; 5 Institute of Womens Health, Fetal and Maternal Medicine, University College London, University of London, London, UK; 6 Centre for Genomics and Child Health, Blizard Institute, Barts and The London School of Medicine, Queen Mary University of London, London, UK; 7 Department of Mathematics and Natural Sciences (MNS), BRAC University, Dhaka 1212, Bangladesh

**Keywords:** Thalassaemia, Awareness, Perceptions, College students, Bangladesh

## Abstract

**Background:**

Thalassaemia is a potentially life-threatening yet preventable inherited hemoglobin disorder. Understanding local socio-cultural context and level of public awareness about thalassaemia is pivotal for selecting effective prevention strategies. This study attempted to assess knowledge and perceptions about thalassaemia among college students in Bangladesh.

**Methods:**

A supervised cross-sectional survey was conducted on 1578 college students using a self-administered structured questionnaire. The survey took place from 15 February 2018 to 17 March 2018 in the Jamalpur district in Bangladesh. Besides the attitude-related questions, the study asked a total of 12 knowledge-related questions, which were scored on a scale of 0–12 points.

**Results:**

Over two-thirds (67%) of the college students had never heard of thalassaemia. The urban-rural dichotomy was observed among those familiar with the term; (46.4% from urban vs. 25.8% from rural colleges). A similar pattern was observed for knowledge score; 5.07 ± 1.87 for students from the urban colleges compared to 3.69 ± 2.23 for rural colleges. Students from the science background had the highest knowledge score (5.03 ± 1.85), while those from arts and humanities background scored lowest (3.66 ± 2.3). Nearly 40% of the students were not sure or did not want to be a friend of a thalassaemia patient. Whereas 39% either declined or remained hesitant about helping thalassaemia patients by donating blood. However, most of the respondents (88%) showed a positive attitude towards ‘premarital’ screening to prevent thalassaemia.

**Conclusions:**

This study has identified critical knowledge gaps and societal misperceptions about thalassaemia. A better understanding of these aspects will be pivotal for disseminating thalassaemia related information. As the first study of this kind in Bangladesh, findings from this study has generated baseline data that would contribute to developing effective intervention strategies in Bangladesh and other countries with a comparable socio-cultural setting.

## Background

Thalassaemia is an inherited hemoglobin disorder which is highly prevalent in Southeast Asia, the Indian subcontinent, Mediterranean and Middle Eastern countries, collectively known as the ‘world thalassaemia belt’ [1, 2]. As a consequence of globalisation, thalassaemia has become a global public health concern. An estimated 1–5% of the world population are carriers of thalassaemia [3] including an estimated 45–70 million people in South Asian countries [4]. Nearly half a million babies are born with serious hemoglobin disorders annually [5], with over 90% of these births in developing countries [3]. In most developing countries the number of thalassemic children is expected to rise in the coming years with the decline in child mortality owing to better management of infectious diseases and malnutrition [4]. In Bangladesh, 6–12% of the population (about 10–19 million people) are carriers of a gene causing thalassaemia [4].

Haematopoietic stem cell transplantation (HSCT) using a matched donor is currently the only curative option for thalassaemia. However, HSCT is expensive, and carries a significant risk of morbidity and mortality. Furthermore, the majority of patients lack a suitably matched donor, and most developing countries lack the required medical resources and expertise to perform HSCT [6]. Standard management of thalassaemia is with regular blood transfusion and iron chelation therapy. In non-subsidised healthcare systems, the cost of standard treatment is high and greatly exceeds the average family income [4]. Moreover, the psychological impact of living with a long-term condition and associated social stigmatization is also a heavy burden for patients and their families in this part of the world [7, 8]. Therefore, prevention is an important goal for developing countries with a high prevalence of thalassaemia.

A number of intervention strategies are implemented in different countries for prevention of thalassaemia. These include mandatory pre-marital screening and genetic counselling (MPSGC), prenatal diagnosis (PND) with an option for termination of affected pregnancy. While some of countries (e.g., Cyprus, Italy, Greece, Turkey and Iran) have achieved high level of success (80–100%) in preventing the births of children with thalassaemia [9]. PND and therapeutic abortion are not routinely offered in some countries, because of cultural and religious restrictions.

Premarital screening should identify at-risk couple and enable them to make informed decisions about their marriage and understand the reproductive options available. However, marriage is a complex affair in conservative Asian cultures. Couples at-risk might still choose to proceed with the marriage if already committed. Calling off a marriage can bring significant social embarrassment or stigma to the couple and their families [10]. This was reflected in a study in Saudi Arabia where implementation of MPSGC did not prevent the majority of at-risk marriages; over 90% of high-risk couples proceeded with the marriage even after receiving genetic counselling [11]. Screening when couples are already committed to their relationship and prior poor awareness about the consequence of having thalassemic children may have been some of the barriers for this outcome in Saudi Arabia [9, 11]. Indeed, a recent study found that nearly half of college students sampled (including 50% of married students) had never heard of thalassaemia despite the implementation of premarital screening in Saudi Arabia [12]. Therefore, raising awareness is an essential component of a successful thalassaemia prevention initiative [13]. Since beliefs, attitudes and perceptions are rooted in culture, any endeavour to raise community awareness requires an understanding of the cultural context. These studies suggest that without mass awaress at community level, the effect of any thalassaemia prevention programme is likely to be diminished.

Bangladesh is a low-income country with a population of over 160 million of whom around 72% live in rural areas. Although situated in the world’s thalassaemia belt, the epidemiology and natural history of thalassaemia in Bangladesh are poorly documented [4]. Unlike, other South Asian countries (India, Pakistan, Sri Lanka and Maldives), there are no organized national programs in Bangladesh for raising awareness, screening carriers or managing patients with thalassaemia [13–17]. PND is not widely offered in Bangladesh, and there are uncertainties about the acceptability of termination of pregnancy. Furthermore, lack of knowledge and misperceptions about thalassaemia are likely to be prevalent.

The long-term focus of raising awareness is critical to pre-marital screening as an intervention. Prior studies in other countries have shown that targeting high school students (age 16 years and above) for thalassaemia education and screening can be effective in preventing the birth of affected children [18, 19]. Therefore, focusing on the post-pubertal age group comprising of high school, college and university students could be part of a future strategy for thalassaemia prevention within the current societal context in Bangladesh.

College-level education is the final stage of schooling for many students, especially women before getting married. According to a recent survey by the Bangladesh Bureau of Statistics (BBS), rural women get married at an average age of 18.3 years and urban women at 19.9 years [20]. Thus, disseminating knowledge on thalassaemia at a younger age is vital. Our study aimed to assess the level of knowledge and perceptions about thalassaemia among college students in Bangladesh.

## Results

### Respondent characteristics

A total of 1578 college students participated in the study. Two thirds (66.7%) of them were females. The majority (64.9%) of the participants had arts and humanities background while 19.9% had science and 15.2% had business studies background. Approximately two thirds of the respondents (65%) were from colleges located in the semi-urban/rural areas (Table 1).

**Table 1:** Demographic characteristics of participants and the proportion of the participants who heard the term/name of thalassaemia (*N* = 1578)

Variables	All participants *N* = 1578 *n* (%)	Have heard of thalassaemia *n* (%)		Pearson’s χ^2^	*p*
		Yes 521 (33%)	No 1057 (67%)		
*Gender*					
Male	526 (33.3%)	171 (32.5%)	355 (67.5%)	0.092	0.777
Female	1052 (66.7%)	350 (33.3%)	702 (66.7%)		
*Discipline studied*					
Science	314 (19.9%)	258 (82.2%)	56 (17.8%)	431.997	< 0.0001
Arts and humanities	1024 (64.9%)	224 (21.9%)	800 (78.1%)		
Business Studies	240 (15.2%)	39 (16.2%)	201 (83.8%)		
*College type*					
Females’ college	453 (28.7%)	194 (42.8%)	259 (57.2%)	27.645	< 0.0001
Co-education	1125 (71.3%)	327 (29.1%)	798 (70.9%)		
Urban	552 (35%)	256 (46.4%)	296 (53.6%)	68.523	< 0.0001
Semi-urban/rural	1026 (65%)	265 (25.8%)	761 (74.2%)		
Private	809 (51.3%)	250 (30.9%)	559 (69.1%)	3.355	0.067
Public	769 (48.7%)	271 (35.2%)	498 (64.8%)		

Over two thirds (67%) of the students reported that they never heard about ‘thalassaemia’. While the majority (82.2%) of the students from the science discipline had heard of thalassaemia (p = < 0.0001), only 21.9 and 16.2% of the respondents with arts and humanities and business studies had heard of thalassaemia respectively (Table 1). The proportion of students who had heard of thalassaemia was nearly twice as high in urban colleges than in semi-urban or rural setting (46.4% vs 25.8% respectively; p = < 0.001). The respondents with science background were more than three times higher in urban colleges compared with rural colleges (35.5% vs 10.5% respectively).

Knowledge and attitude were assessed among 521 (33%) respondents who were familiar with the word/terminology, ‘thalassaemia’. The most frequently mentioned source of information about thalassaemia was textbooks (66.2%) (Additional file 1). Nearly 82% of these participants were from science discipline (Table 1). Other information sources included friends (4.6%), relatives (5.6%), internet resources (3.1%), and doctors (1.7%); 12.5% couldn’t specify the source (Additional file 1).

### Knowledge

Table 2 reports knowledge about thalassaemia in the study population. More than half of the respondents (56%) correctly answered that thalassaemia is a genetic disease while nearly one third (29.8%) did not have any idea. However, over 50% of participants responded that thalassaemia is a contagious disease As such, some of the respondents who identified thalassaemia as a genetic disease also believed the nature of its contagiousness (11.1%). We hypothesize, this could be an unintended result of emphasis given to communicating the chance of blood-borne contaminations (e.g., HIV, HBV, HCV, etc.) in the mass media. Regarding risk factor of thalassaemia, about 64% did not know that thalassaemia was not a transfusion-transmitted disease. Likewise, 60.4% of respondents correctly answered that marriage between two carriers could result in the birth of a thalassaemic child. Only 11.9% responded ‘NO’ when asked whether there is a chance of having a child with thalassaemia disease if one parent is a carrier. While consanguinity is a well-known risk factor for thalassaemia, nearly half of our respondents (48.9%) didn’t know about this fact, and 29% didn’t even think of it as a risk factor. Nearly 26% of students believed that thalassaemia is a preventable disease, and 24.7% thought that thalassaemia is a curable disease. About 63% of the respondents were aware that they could be a carrier, and 65.2% knew that thalassaemia can be detected by blood test.

**Table 2:** Distribution of responses (correct/incorrect/doesn’t know) of 12 knowledge related questions among students who have heard about thalassaemia (*n* = 521)

Questions	Correct n (%)	Incorrect n (%)	Don’t know n (%)	χ2	p
*1. Thalassaemia is a contagious disease (NO)*	271 (52%)	106 (20.3%)	144 (27.7%)	79.84	< 0.0001
Arts and humanities	72 (13.8%)	58 (11.1%)	94 (18%)		
Business Studies	15 (2.9%)	12 (2.3%)	12 (2.3%)		
Science	184 (35.5%)	36 (6.9%)	38 (7.4%)		
*2. Thalassaemia is a genetic disease (Yes)*	292 (56%)	74 (14.2%)	155 (29.8%)	28.26	< 0.0001
Arts and humanities	99 (19%)	33 (6.3%)	92 (17.7%)		
Business Studies	22 (4.2%)	6 (1.2%)	11 (2.1%)		
Science	171 (32.8%)	35 (6.7%)	52 (10%)		
*3. Thalassaemia could be transmitted through blood transfusion from a person with thalassaemia (No)*	43 (8.3%)	336 (64.4%)	142 (27.3%)	34.93	< 0.0001
Arts and humanities	23 (4.4%)	114 (21.9%)	87 (16.7%)		
Business Studies	3 (0.6%)	25 (4.8%)	11 (2.1%)		
Science	17 (3.3%)	197 (37.8%)	44 (8.4%)		
*4. Marriage between two carriers can lead to a child with thalassaemia major (Yes)*	315 (60.4%)	42 (8.1%)	164 (31.5%)	32.85	< 0.0001
Arts and humanities	107 (20.5%)	19 (3.6%)	98 (18.8%)		
Business Studies	22 (4.2%)	4 (0.8%)	13 (2.5%)		
Science	186 (35.7%)	19 (3.6%)	53 (10.2%)		
*5. If one parent is a carrier, the couple has a chance of having a child with thalassaemia disease (No)*	62 (11.9%)	285 (54.7%)	174 (33.4%)	38.31	< 0.0001
Arts and humanities	29 (5.6%)	93 (17.9%)	102 (19.6%)		
Business Studies	7 (1.3%)	17 (3.3%)	15 (2.9%)		
Science	26 (5%)	175 (33.6%)	57 (10.9%)		
*6. Marriage between close relatives can increase the chance of thalassaemia (Yes)*	115 (22.1%)	151 (29%)	255 (48.9%)	3.0	0.588
Arts and humanities	44 (8.4%)	63 (12.1%)	117 (22.5%)		
Business Studies	7 (1.3%)	13 (2.5%)	19 (3.6%)		
Science	64 (12.3%)	75 (14.4%)	119 (22.8%)		
*7. Thalassaemia carriers are as healthy as normal people (Yes)*	184 (35.3%)	163 (31.3%)	174 (33.4%)	22.512	< 0.0001
Arts and humanities	79 (15.2%)	50 (9.6%)	95 (18.2%)		
Business Studies	11 (2.1%)	13 (2.5%)	15 (2.9%)		
Science	94 (18%)	100 (19.2%)	64 (12.3%)		
*8. Thalassaemia is a preventable disease (Yes)*	134 (25.7%)	165 (31.7%)	222 (42.6%)	14.79	0.005
Arts and humanities	60 (11.5%)	55 (10.6%)	109 (20.9%)		
Business Studies	14 (2.7%)	9 (1.7%)	16 (3.1%)		
Science	60 (11.5%)	101 (19.4%)	97 (18.6%)		
*9. Thalassaemia is a completely curable disease (No)*	129 (24.7%)	201 (38.6%)	191 (36.7%)	20.30	< 0.0001
Arts and humanities	42 (8.1%)	93 (17.9%)	89 (17.1%)		
Business Studies	4 (0.8%)	23 (4.4%)	12 (2.3%)		
Science	83 (15.9%)	85 (16.3%)	90 (17.3)		
*10. Which part of the human body or organ is affected by Thalassaemia? (Blood or circulatory system)*	61 (11.7%)	26 (5%)	434 (83.3%)	23.82	< 0.0001
Arts and humanities	12 (2.3%)	8 (1.5%)	204 (39.2%)		
Business Studies	2 (0.4%)	2 (0.4%)	35 (6.7%)		
Science	47 (9%)	16 (3.1%)	195 (37.4%)		
*11. Anyone could be a thalassaemia carrier including you. (Yes)*	330 (63.4%)	118 (22.6%)	73 (14%)	17.63	0.001
Arts and humanities	123 (23.6%)	55 (10.6%)	46 (8.8%)		
Business Studies	27 (5.2%)	7 (1.3%)	5 (1%)		
Science	180 (34.5%)	56 (10.7%)	22 (4.2%)		
*12. Thalassaemia can be identified by blood test (Yes)*	340 (65.2%)	113 (21.7%)	68 (13.1%)	17.16	0.002
Arts and humanities	129 (24.8%)	52 (10%)	43 (8.3%)		
Business Studies	24 (4.6%)	10 (1.9%)	5 (1%)		
Science	187 (35.9%)	51 (9.8%)	20 (3.8%)		

Table 3 reports the association between socio-demographic variables and level of knowledge about thalassaemia among the study population. Out of a total possible score of 12, the mean knowledge score was very poor (4.73 ± 1.54). The knowledge score was not significantly different across gender groups. However, knowledge scores varied significantly by academic disciplines. Respondents with science background had the highest knowledge score (5.03 ± 1.85) while the lowest score was reported for students with arts and humanities background (3.66 ± 2.3). A significant difference in knowledge scores was observed among the students from urban (5.07 ± 1.87) and rural colleges (3.69 ± 2.23). Despite some aspects being better known compared to others, the maximum number of correct answers did not exceed two-thirds of the questions.

**Table 3:** Association between demographic variables and total thalassaemia knowledge scores (*n*=521)

Variables	Total score (Mean ± SD)	*Kruskal-Wallis* *p*-value
*Gender*		
Male	4.35 ± 1.89	0.728
Female	4.38 ± 2.29	
*Major*		
Arts and humanities	3.66 ± 2.3	< 0.0001
Science	5.03 ± 1.85	
Business Studies	4.05 ± 1.97	
*College type*		
Females’ college	4.22 ± 2.14	0.236
Co-education	4.46 ± 2.18	
Urban	5.07 ± 1.87	< 0.0001
Semi-urban/rural	3.69 ± 2.23	
Public	5.17 ± 1.85	< 0.0001
Private	3.50 ± 2.16	

*SD* Standard deviation,

Total score of thalassaemia knowledge was 12

### Attitude

Table 4 reports the attitudes of the study population towards thalassaemia. Overall, a higher proportion of the students who heard of thalassaemia showed positive attitudes towards thalassaemia in response to the questionnaire. The majority of the students (88.1%) would prefer premarital screening to prevent thalassaemia. Approximately 60.8% were positive about donating blood to thalassaemia patients. Most of the respondents (90.4%) were willing to spread awareness about thalassaemia in the community. Likewise, 90% were of the opinion that their educational institution should educate them on thalassaemia. When asked whether they would be a friend of a thalassaemia patient, only 58.9% of the students agreed. There was a significant difference in attitudes between students from urban colleges (5.19 ± 1.22) and semi-urban/rural colleges (4.27 ± 1.24) (Additional file 2).

**Table 4:** Attitudes towards thalassaemia among students who had heard about thalassaemia (*n* = 521)

Questions or Actions	Positive n (%)	Neutral n (%)	Negative n (%)
1. I would take the necessary blood test before marriage to prevent the birth of a thalassaemic child	459 (88.1%)	59 (11.3%)	3 (0.6%)
2. I would like to donate my blood for patients with thalassaemia	317 (60.8%)	129 (24.8%)	75 (14.4%)
3. I would be happy to befriend a patient with thalassaemia	307 (58.9%)	124 (23.8%)	90 (17.3%)
4. I would like to inform others about the potential danger of thalassaemia	471 (90.4%)	48 (9.2%)	2 (0.4%)
5. I would take necessary steps to ensure blood testing for thalassaemia before the marriage of my family members	441 (84.6%)	78 (15%)	2 (0.4%)
6. I want my school to take the initiative to generate awareness among students about thalassaemia	469 (90%)	52 (10%)	0 (0%)

## Discussion

The purpose of this study was to assess knowledge and perceptions regarding thalassaemia among college students. This is the first study of its kind (apart from the study of parents of individuals with thalasseemia [21]) in Bangladesh which has revealed substantial knowledge gaps and misconceptions about thalassaemia.

### Poor knowledge and attitude

We found that the level of thalassaemia awareness was surprisingly low, and only one-third of the respondents (33%, *n* = 1578) had heard of thalassaemia. Being situated in the world’s thalassaemia belt with an estimated carrier of about 6–12%, this result is quite unexpected [4] as compared to other thalassaemia-prevalent countries such as Malaysia (~ 87%), Italy (85%), Greece (95%), Bahrain (65%), Turkey (58%) and Saudi Arabia (48%) [12, 22–26]. This indicates that a major public health concern has been overlooked in Bangladesh.

While a large number of participants did not know anything about thalassaemia, the overall knowledge (represented by knowledge scores) of the participants who declared to know about the disease, was not satisfactory. Nearly two-third (64.5%) of the respondents who had heard of thalassaemia were not aware of the fact that thalassaemia carriers are essentially as healthy as non-carriers. Similar lack of awareness towards thalassaemia carriers has been reported from Saudi Arabia (64%), India (~ 89%) and Malaysia (79.7%) [12, 26, 27]. This widespread lack of knowledge might be a pointer towards other socio-cultural problems which make life more difficult not only for patients with thalassaemia but for thalassaemia carriers as well. This is particularly concerning because of the potential for those who think they know about thalassaemia to pass on incorrect information to others who don’t know about this disease. In South Asian cultures, particularly in Bengali society, blood, marriage, kinship, identity and parenthood are highly valued and therefore, genetic mutation or thalassaemia carrier status could be equivalent to corrupting blood (Rokter dosh) and thus might be subjected to stigmatization [28]. In Bangladesh (and the South Asian region overall), societal perceptions about the value of the potential bride and groom play a vital role in approving a marriage. Hence, any negative attitude about thalassaemia patients or carriers might seriously affect their social and personal life as a result of stigmatization of individuals or even entire families.

With overall poor knowledge, a significant proportion of the respondents had a negative attitude towards thalassaemia as a disease. We found that approximately 40% of the students stayed neutral or did not want to be friends with thalassaemia patients while the same proportion (~ 39%) either declined or remained hesitant about helping thalassaemia patients by donating blood. The implication of this negative attitude is perhaps much larger than thalassaemia patients merely losing some friends. Arguably, if the educated community which is aware of thalassaemia cannot accept patients as their friends, it is likely that stigmatization of the patients would be more widespread in the rest of the community. This might indicate a serious issue of fear or stigma associated with thalassaemia patients and thalassaemia as a disease in general.

The worst consequence of this dangerous combination of lack of knowledge and misperception could make people fearful of being identified as a carrier. This was perhaps evident from the fact that 12% of respondents did not agree to get screened before marriage. With such reservations prevailing among the college-going educated community, it is possible that the effects of a negative attitude towards thalassaemia as a disease would also be extended to carriers. As such, any attempt to prevent thalassaemia might face a challenge from the community itself; potential responses could include- refusal to undergo screening tests, presenting obstacle to efforts encouraging screening, repurcussions against the idea of preventing marriage between two thalassaemia carreers, etc. The main reasons behind such negative reactions could include fear of stigmatization (e.g., the notion of having blood line tainted which could potentially lead to enitre family or sect getting socially isolated) and fear of violating divine plan.

### Dissemination

The difficulty of disseminating health information at the community level has also been highlighted in the present study. Contrary to expectation, this study has revealed that thalassaemia-related information spread via mass media (newspaper, TV, Radio and internet) and social media (i.e., Facebook) were perhaps insufficient (only 6% respondents identified these as knowledge-source). Approximately 92 million people in Bangladesh have access to the internet [29] and nearly one third (25–30 million) of them use social media, (particularly Facebook), which is very popular among students aged 18–24 [30, 31]. Among other sources, medical doctors contributed little (~ 1.7%) to generating awareness about thalassaemia in Bangladesh. In countries with successful thalassaemia prevention program, physicians (family doctors, obstetricians, and genetic counsellors) have played a significant role in disseminating thalassaemia related information. For instance, nearly 70% of the target population of Sardinia were informed by physicians [13]. Similarly, a significant proportion of the general population in Italy (15%) and Malaysia (9%) were familiar with thalassaemia through physicians [23, 26]. However, the scenario in the study setting, and perhaps similarly for the rest of Bangladesh, is remarkably different. Such a low contribution of physicians for dissemination might be due to scarcity of registered physicians in the rural areas, weak patient-physician relationship, and short consultation time (approximately 1–3 min) given to each patient sometimes due to sheer high number of paitients to be attended by each physician [32, 33]. Thus, the means for  educating and engaging healthcare professionals (physicians, health co-workers) in raising thalassaemia-awareness in Bangladesh needs to be reviewed.

For awareness, sending short messages (SMS) to the mobile phone users is being practiced by the Government of Bangladesh (GoB) as there are over 157 million mobile phone subscribers. About one month before conducting our survey the GoB sent an SMS to all mobile phone users on the eve of the first government-sponsored thalassaemia initiative to raise thalassaemia awareness. Despite this effort which was likely to reach a large target population, our study has revealed that more than two-third (67.5%) of college students (the majority of them are expected to be phone users) had not heard about thalassaemia. Although the question (whether the students wereaware of the SMS) was not included in the questionnaire, more than two-third respondents remaining unaware of thalassaemia suggests that disseminating health-related information at the community level using this approach needs to be reviewed to enhance effectiveness.

In our study, the curriculum was found to be the most effective source (~ 62.6%) of information for students who heard about thalassaemia. Amongst the three disciplines, science students were only taught about thalassaemia in their curriculum in grade XI and XII. This education includes classroom discussion by their teachers and self-reading. Thus, the inclusion of thalassaemia-related education in the curriculum for arts and humanities and business studies students might be effective for thalassaemia awareness. Thalassaemia related education as a part of the school curriculum has contributed to successful prevention programs around the world including Sardinia, Italy, Iran [13]. If the college students are targeted, their teachers could then serve as a source of information for the rest of the community. However, nearly one-fifth of the students (17.8%) from science discipline had not heard about thalassaemia, suggesting that inclusion of teaching about thalassaemia in the curriculum by itself might not be sufficient. In this regard, classroom-based discussion among friends could be of great potential as relatives/friends (~ 10%) have been reported to be a good source of thalassaemia-related knowledge.

The proportion of students studying science has declined from 42% in 1990 to 22% in 2015 [34]. In rural areas of Bangladesh (where about 70% of the population live), the majority of the collegestudents are enrolled in arts and humanities or business studies. This was also observed in our study. Therefore, incorporation of basic thalassaemia related information in the curricula of all three disciplines is recommended.

Despite many challenges revealed by this study, almost all of the college-students (> 90%) who heard about thalassaemia were keen to enhance awareness and expected their colleges to take the initiative in education about thalassaemia. The higher score on attitude implies that the college students were really feeling empathy yet they were not familiar with basic facts about thalassaemia. This highlights a positive inclination which could be exploited for intervention.

Although the attitude towards thalassaemia or to patients is not directly dependent on the knowledge about it, our survey also found that the discipline studied was a significant determinant of perception; the attitude of the students from science background was significantly more positive compared to those studying arts and humanities or business studies. This reinforces the importance of appropriate education incorporated within the curriculum.

Although the levels of perception varied between students from urban and rural colleges, this variation was mostly due to the urban colleges having higher proportion of students from science background. This could be an indication of disparity among the private and public colleges. Since public colleges (esp. outside of the capital city, Dhaka) traditionally attract better quality students and teachers. As such the overall environment is perhaps more conducive for better health literacy.

A high proportion of participants (88%) responded that they would get screened for thalassaemia with a blood test before marriage. Previous studies in Canada and India showed that high-school students had a high level of interest for thalassaemia screening with a participation rate of approximately 80% after attending educative programmes [19]. This positive attitude should be taken into consideration before adopting any national policy.

Thalassaemia is becoming a higher priority for health planning in Bangladesh. The Ministry of Health has drafted a management guideline for medical practitioners, and organized national and international workshops [35]. The Minister of Health also announced that “Bangladesh would be thalassaemia free by 2028”. Government efforts to curb thalassaemia has emphasized so far on providing partial financial support, improving clinical management of patients, and raising public awareness. However, due to financial and logistic constraints, government initiatives often are inadequate. As a public interest litigation issue, a writ petition has been submitted to the High Court of Bangladesh to make premarital screening mandatory, which has attracted public attention [36]. This study complements these initiatives and has identified critical knowledge gaps, societal misperceptions and effective means of disseminating thalassaemia related information targeting an important age group (college students). Our findings would contribute to developing a policy for effective thalassaemia awareness, screening and intervention strategies (e.g., disseminating knowledge, raising thalassaemia awareness, encourage screening, etc.) in developing countries like Bangladesh.

### Limitations

Since no validated questionnaire was available for testing knowledge and attitude regarding thalassaemia, the questions asked in this study were based on studies conducted in comparable socio-cultural backgrounds. General limitations of this study are those applicable to cross-sectional studies conducted by self-administered questionnaire. With regard to the sample population our study sample is representative to majority of the districts (33 out of 64) in Bangladesh considering demographic and socio-economic factors [37], but may not be fully generalizable to the population of Bangladesh as a whole.

## Conclusions

In a predominantly illiterate society/community, a specific strategy is required to improve the health-literacy among the public. The socio-economic and resource-limited nature of the large part of Bangladesh (especially the peripheral districts) often renders the conventional media ineffective in this regard, and this has been confirmed in our study.

Highlights of the study come from the fact that majority of the college students were unfamiliar with the term ‘thalassaemia’, knowledge towards this disease was poor, and attitude towards thalassaemia carriers or patients was negative to some extent. Hence, making the college students aware of the risks of thalassaemia and motivating them to get screened for the disease demands a significant cultural shift. While engaging representatives from all levels of the community might be needed, college-teachers could be very effective resources in this regard as they have a good connection with all the levels in the community. Insights gained from this study have highlighed the necessity of having comprehensive understanding of the sociocultural conditions before proposing or selecting interventions strategies. Overall, this report will substantially contribut to prevent spread of thalassaemia in resource limited conditions like Bangladesh

## Methods

### Study setting

A cross-sectional study was conducted in Jamalpur district (one of the 64 districts in Bangladesh). Jamalpur has a population of 2.3 million (2011 census) and is located 140 km northwest of the capital city, Dhaka. Bangladesh is generally homogeneous in the in terms of demographic and socio-cultural factors [37]. For instance, considering socio-economic factors our study setting (Jamalpur) is representative of 33 districts within Bangladesh [37].

### Sampling

The total size of the target population at intermediate college level (the equivalent of grades 11–12 in the USA) in Jamalpur was 4505 [38]. For an accuracy level of 95% with a confidence interval of ±3.0% the sample size was calculated to be 863 [39]. For sampling, four sub-districts (or Upazila) out of seven were selected randomly. Considering the relative population size of the study sub-districts, and in order to exceed the minimum sample size, eleven intermediate colleges were selected from the list of 25 colleges using a random number table. The final list comprised of three colleges from urban, five from semi-urban, and three from rural settings. Among these 11 colleges, eight were co-educational while three were female-only.

Pre-trained data collectors conducted surveys from 15 February 2018 to 17 March 2018. From each college, three classrooms were randomly selected to include participants with the background of science, arts and humanities, and business studies. Students were not notified in advance about the survey. Since prior familiarity with thalassaemia as a disease (i.e., the term ‘thalassaemia’) was a critical component of our study, we ensured that the word thalassaemia was not mentioned in the invitation to, and preparations for the survey. Instead, on the day of the survey, a general announcement was made in the classrooms that a survey on a health-related issue would take place. Interested students were requested to gather in a classroom were included in the study. Their responses were recorded using a self-administered questionnaire in the presence of the class teachers. A total of seventeen (1%) incompletely filled questionnaires were excluded from this study.

### Instrument

Data on knowledge and attitude about thalassaemia were collected using a structured questionnaire. The design of the study questionnaire was based on a review of studies found in the literature [12, 25–27, 40], and after consultation with experts including clinicians, public health researchers and a statistician. The questionnaire, which consisted of 22 questions divided into 2 parts, was prepared in the Bengali language, however, some of the medical terminologies could not be directly translated. Notably, ‘thalassaemia’ was a direct translation into Bengali to refer to the disease. The absence of any alternative native expressions of this disease was reaffirmed by consulting with patients, local clinicians, and community leaders. The questionnaire was piloted on 20 undergraduate students and their feedback were included in the final questionnaire. A team of trained data enumerators administered the questionnaire after obtaining written approval from the respective college authority.

Printed version of the questionnaire contained two pages. On the first page, consent and demographic information on the participants (name of the college and study discipline) were documented. Then the participants were asked if they had heard of “thalassaemia”. Those who answered “yes” were asked to proceed with the individual questions. The participants’ knowledge about thalassaemia, its carrier state and prevention was assessed using 12 items. Of these, responses to 9 items were recorded using a 5-points Likert scale (strongly agreed, agreed, don’t know, disagreed and strongly disagreed). Attitudes towards thalassaemia were then assessed, including views on premarital screening, relationships and blood donation to thalassaemia major patients, raising awareness personally and through their educational institutions. Two other questions specifically addressed opinions on factors influencing the choice of future partners and the most effective media to develop thalassaemia awareness. For both questions, responses were recorded from multiple given answers.

### Statistical analyses

Descriptive and inferential statistical procedures were used to analyze the data. Categorical variables were presented using counts and percentages, and continuous variables were summarized using means and standard deviations. For testing the association between categorical variables, Pearson’s chi-square test with continuity correction was used when necessary. The Likert scale measurements were summarized by computing the total scores. Knowledge score was calculated on a scale where minimum and maximum attainable scores were 0 and 12. Knowledge was considered satisfactory when the score was equal or more than median attained score. Similar scoring system (0–6 scale) was used for attitude related questions. Means and standard deviations were calculated for the scores. For continuous variables, normality tests were performed first, and if needed, nonparametric Kruskal-Wallis one way ANOVA tests were performed. A *p*-value smaller than 0.05 was considered significant.

## Supplementary information

**Additional file 1.** Major sources of information regarding Thalassaemia.

**Additional file 2. **Distribution of positive attitudes among students of different colleges (*N* = 521) based on 6 Thalassaemia attitudes/perceptions questions.

## Data Availability

Data generated in this study are not publicly available. Only aggregate summary have been provided in the manuscript.
