# Supplementary material for: Lack of knowledge and misperceptions about thalassaemia among college students in Bangladesh: a cross-sectional baseline study
Source: Orphanet J Rare Dis. 2020 Feb 21;15:54. doi: 10.1186/s13023-020-1323-y (PMC7035777; doi:10.1186/s13023-020-1323-y)
Supplement: Supplementary file 1 — Additional file 1. Major sources of information regarding Thalassaemia. [file 13023_2020_1323_MOESM1_ESM.docx]

Additional file 1. Major sources of information regarding Thalassemia

| **Source** | **n** | **%** |
| --- | --- | --- |
| Curricula | 345 | 66.2% |
| Relative | 29 | 5.6% |
| Friends | 24 | 4.6% |
| Internet | 16 | 3.1% |
| Facebook | 3 | 0.6% |
| TV | 10 | 1.9% |
| Doctor | 9 | 1.7% |
| Newspaper | 2 | 0.4% |
| Radio | 1 | 0.2% |
| Unknown | 65 | 12.5% |
