# Supplementary material for: Lack of knowledge and misperceptions about thalassaemia among college students in Bangladesh: a cross-sectional baseline study
Source: Orphanet J Rare Dis. 2020 Feb 21;15:54. doi: 10.1186/s13023-020-1323-y (PMC7035777; doi:10.1186/s13023-020-1323-y)
Supplement: Supplementary file 2 — Additional file 2. Distribution of positive attitudes among students of different colleges (N = 521) based on 6 Thalassaemia attitudes/perceptions questions. [file 13023_2020_1323_MOESM2_ESM.docx]

Additional file 2. Distribution of positive attitudes among students of different colleges (N=521) based on 6 Thalassemia attitudes/perceptions questions.

| Variables | Mean ± SD | *Kruskal-Wallis p* value |
| --- | --- | --- |
| *Gender* | | |
| Male | 4.6±1.53 | 0.065 |
| Female | 4.78±1.54 |  |
| *Major* | | |
| Arts and humanities | 4.22±1.64 | <0.0001 |
| Science | 5.19±1.24 |  |
| Business Studies | 4.51±1.76 |  |
| Others |  |  |
| *College type* | | |
| Females’ college | 4.59±1.80 | 0.944 |
| Co-education | 4.80±1.35 |  |
|  |  |  |
| Urban | 5.19±1.22 | <0.0001 |
| Semi-urban/rural | 4.27±1.67 |  |
|  |  |  |
| Public | 5.14±1.24 | <0.0001 |
| Private | 4.28±1.70 |  |
